# Persian Walnut (*Juglans regia* L.) Bud Dormancy Dynamics in Northern Patagonia, Argentina

**DOI:** 10.3389/fpls.2021.803878

**Published:** 2022-02-03

**Authors:** Ricardo Alfredo del Barrio, Gustavo Adolfo Orioli, Andrea Soledad Brendel, Lilia Ivone Lindström, Cecilia Noemí Pellegrini, José Antonio Campoy

**Affiliations:** ^1^Sede Atlántica, Universidad Nacional de Río Negro, Viedma, Argentina; ^2^Departamento de Agronomía, Universidad Nacional del Sur, Bahía Blanca, Argentina; ^3^Univ. Bordeaux, INRAE, Biologie du Fruit et Pathologie, UMR 1332, Villenave-d’Ornon, France; ^4^Department of Chromosome Biology, Max Planck Institute for Plant Breeding Research, Cologne, Germany

**Keywords:** bud development, bud dormancy, carbohydrates, chill requirement, heat requirement, microscopy, nut trees

## Abstract

Temperate deciduous fruit trees survive winter temperatures by entering a dormant phase in their aerial meristematic organs. Release from bud dormancy occurs after chill requirements (CR) have been satisfied, whereas bud burst/flowering follows heat requirement (HR) fulfillment. The physiological basis behind these metrics remains elusive. In this study, we are presenting the first multidisciplinary dormancy progression analysis in northern Patagonia, linking (1) forcing/field phenology, (2) bud anatomical development, and (3) soluble sugar (sucrose, glucose, and fructose) dynamics in *Juglans regia* L. CR and HR were determined for ‘Chandler’ and ‘Franquette,’ two walnut cultivars with markedly different CR, in artificial chill/forced heat trials (three seasons) and in-field chill/forced heat tests (five seasons) using excised twigs either with or without apical buds (non-decapitated and decapitated). The soluble sugar dynamics of ‘Chandler’ (high-performance liquid chromatography) and the anatomical changes of the buds (light microscopy) of the two cultivars were analyzed during endo-ecodormancy progression in one and two seasons, respectively. The CR defined by artificial chill tests proved to be an overestimation compared to the field determinations. Moreover, HR was the main driver in the phenology dynamics, as expected for a high-chill region. ‘Chandler’ showed an average of 10.3 field chill portions (CP) and 2,163 Growing Degree Hours (GDH°C) less than ‘Franquette’ for dormancy release and bud burst, respectively. These results were consistent with the transition of the shoot apex from the vegetative to the reproductive phase and the soluble sugar profile. The decrease in sucrose between 15 and 30 days after CR fulfillment could be a reliable biological marker for endodormancy release in walnut, while the increase in fructose and glucose is likely an osmolyte and cellulosic carbon source in pre-sprouting. In addition, we discuss the effect of paradormancy thanks to our apical bud experiment (with or without). Our results improve the current understanding of endo-ecodormancy progression in walnut and provide insightful results for walnut production (i.e., cultivation practices such as pruning) as well as for further application in dormancy modeling, to infer the ideotypes that should be bred for future climate conditions.

## Introduction

In the progression from late summer and early fall to winter, temperate tree species go through a dormancy stage to survive unfavorable thermal conditions (Campoy et al., 2011b; Legave et al., 2013; Fadón and Rodrigo, 2018). Previous studies have revealed that this process is mainly regulated by thermal and photoperiodic conditions (Basler and Körner, 2012, 2014; Singh et al., 2017) and that thermal conditions are central to bud dormancy and bud burst development in different fruit tree species (Egea et al., 2003; Heide, 2008, 2011; Ghelardini et al., 2010; Guo et al., 2015; Benmoussa et al., 2017a).

An example of phenology-environment relationships, fruit trees growing in temperate and cold temperate zones require winter chill to overcome dormancy. During the physiological stage known as endodormancy, buds remain in lethargy as a result of their physiological conditions regardless of favorable growth conditions. Endodormancy release is followed by *quiescence* (ecodormancy), a period during which buds remain dormant as a result of unfavorable environmental conditions (Lang et al., 1987; Considine and Considine, 2016). The thermal needs in each of these phases can be expressed as chill requirements (CR) and heat requirements (HR) for endo- and ecodormancy, respectively (Luedeling, 2012).

Although the study of the chill and heat requirements for budburst and flowering in deciduous fruit trees dates back into the last two centuries (Knight, 1801; Coville, 1920), it has been more extensively researched in different crops in recent decades (De Melo-Abreu et al., 2004; Andreani et al., 2014; Alonso et al., 2017; Benmoussa et al., 2017b; Anzanello et al., 2018; Ruiz et al., 2018). Despite recent milestones in our understanding of bud dormancy control in model trees (Singh et al., 2019; Azeez et al., 2021), gaps still remain. This is especially true of temperate fruit and nut trees since the interactions among the different biological processes and mechanisms involved are not yet fully understood (for review refer to Beauvieux et al., 2018; Lloret et al., 2018; Falavigna et al., 2019; Liu and Sherif, 2019; Yang et al., 2021).

In addition, the phenology models developed from the chill and heat requirements of different deciduous fruit tree species fit certain climate conditions, but as of yet, there are no general models that cover interregional environmental differences (Luedeling et al., 2009b,c), although several researchers have recently made significant progress in this regard (i.e., Chmielewski and Götz, 2016; Darbyshire et al., 2017; Fernandez et al., 2021). A thorough understanding of the dormancy process and its release is key to optimizing the geographical distribution of a given species (Alburquerque et al., 2008; Luedeling et al., 2011; Campoy et al., 2019; Rodríguez et al., 2019) and for correctly anticipating spring phenological responses to future climate change (Wang et al., 2020).

The importance of these factors in the production of deciduous fruit trees, such as walnuts, is well known (Chandler et al., 1937; Mauget, 1976; Mauget and Germain, 1980; Charrier et al., 2011, 2018). The winter chill is an agroclimatic driver that integrates the length of cold periods as well as prevailing temperature ranges. Low chill availability contributes to irregular, delayed, and asynchronous growth, flowering, and fruit development in the following growing season (Erez, 2013; Melke, 2015). At the same time, flowering must occur late enough to avoid spring frosts, but not so late that it results in a failed growing season (Kramer et al., 2017). Moreover, the chill and heat requirements of a cultivar/species must suit the climate of the growing area to ensure a successful fruit production throughout the orchard cycle (Guo et al., 2014; Díez-Palet et al., 2019). In the context of climate change, the success of fruit orchards (as a long-term investment) requires precise planning and relies on our capacity to predict orchard performance in future climate scenarios (Luedeling et al., 2011; Rodríguez et al., 2021).

Chill requirements have been identified as necessary for dormancy breaking and subsequent budburst and flowering. This metric is considered specific for each species and cultivar or variety and its utility depends on our ability to predict their adaptability to a particular environment (for review refer to Luedeling, 2012). The experimental estimation of the CRs for the different walnut cultivars as well as of chill availability in given geographical locations is therefore pivotal in temperate species research in general (Luedeling et al., 2009b).

In the present study, we used controlled environment procedures to quantify the chill and heat requirements of fruit trees using excised twigs and two methodological approaches. The first involved cutting the plant material (twigs) and storing it in a cold device simulating different chill accumulations to stimulate dormancy release and then forcing warming until bud burst (Gariglio et al., 2006; Campoy et al., 2013; Hussain et al., 2015). The second approach consisted of excising the plant material periodically so that chill accumulated in the field and then forcing heat accumulation under controlled conditions (Razavi et al., 2011; Campoy et al., 2012; Measham et al., 2017). Okie and Blackburn (2011) combined both methods to achieve better chill and heat requirement estimations.

Detailed microscopy observations of the anatomical and morphological changes that occur within buds before any available visible signs are very helpful for understanding dormancy progression (Sutinen et al., 2009). To our knowledge, the first documented studies on the morphology of vegetative and floral buds of *Juglans regia* L. were conducted by De Candolle (1862) as cited by Nast (1935). Several researchers have subsequently studied the anatomy of walnut buds, especially staminate ones (Luza and Polito, 1988; Polito and Pinney, 1997; Polito, 1998; Germain et al., 1999; Sabatier and Barthélémy, 2001; Sabatier et al., 2003; Li et al., 2011; Gao et al., 2012, 2014). The temporal sequencing of anatomical and morphological changes in walnut buds varies according to the cultivar and the climatic conditions (Zhang et al., 1995; Polito and Pinney, 1997). To date, no studies have been conducted in walnut cultivars growing in northern Patagonia.

Anatomical changes in buds are accompanied by modifications in the carbon metabolism throughout the leafless period (Poirier et al., 2010). In response to low temperatures in winter, starch degraded by amylases is used for sucrose synthesis. In reserve tissues, sucrose is produced and then transported *via* the xylem to buds, where it is hydrolyzed to glucose and fructose to produce energy and carbonic precursors (Yoshioka et al., 1988). Until the formation of an efficient photosynthetic apparatus in spring, buds grow mainly at the expense of carbohydrate reserves (Decourteix et al., 2008). Dormancy release and bud burst progression could thus be described from a trophic perspective, i.e., related to sugar dynamics. Sucrose is the most common transport sugar and can be hydrolyzed to hexoses (glucose and fructose) (Decourteix et al., 2008; Charrier et al., 2018). Unveiling the relationships between bud anatomy and morphology and the physiological and trophic processes involved in them still requires research in walnut (Gao et al., 2014).

The main goal of this study was to perform the first comprehensive study of dormancy progression linking forcing experiments, bud development, and soluble sugar in the climatic conditions of northern Patagonia, Argentina. We characterized the dormancy progression using artificial and field chill experiments, which were carried out for 3 and 5 years, respectively, to account for year-by-year variability. We complemented the forcing experiment with an in-depth study of the anatomical development of buds of two reference walnut cultivars, ‘Chandler’ and ‘Franquette,’ with markedly different CR. Finally, we enhanced this study by evaluating the soluble sugar (sucrose, glucose, and fructose) dynamics in ‘Chandler’ and their relationship with the physiological and anatomical progression of dormancy in walnut.

## Materials and Methods

### Study Site and Cultivars

This study was carried out in the Agricultural Experimental Station (EEAVI, Argentine National Agriculture Technological Institute -INTA, Province of Río Negro) in the lower valley of the Black River (“Río Negro” in Spanish), located at 40°49′ SL., 63°04′ WL. and 4 m.a.s.l. The annual mean temperature in the area is 14.1°C and the winters are cold-temperate.

Excised shoots of *J. regia* cultivars ‘Chandler’ (a medium-chill Californian cultivar) and ‘Franquette’ (a high-chill French cultivar), belonging to the EEAVI walnut collection, were used for this study. The trees were 7 years old at the beginning of the trials. They were planted at commercial density (7 m × 5 m), grafted on *J. regia* L. domestic seedling rootstock, in loamy soils, and they were well-nourished using furrow or mantle irrigation. These two cultivars span a wide range of flowering time in this species and represent 95% of the production in the Black River growing area.

‘Chandler’ is a highly productive cultivar (90% of lateral buds are fruitful), and the trees are moderately vigorous and semi-upright. It was obtained by the University of California, Davis (United States) breeding program from the cross ‘Pedro’ x ‘UC 56-224.’ ‘Chandler’ walnuts are harvested mid to late season. The nut size is large, and the nut shape is oval and smooth with 90–100% light kernels. The kernel percentage is 49%. ‘Chandler’ usually has only moderate blight problems. The ‘Franquette’ cultivar, on the other hand, is originally from the Isère River Valley (France). ‘Franquette’ trees are large and upright with moderate to high vigor. This cultivar is a little less productive than ‘Chandler’: mainly the terminal buds are fruitful, in addition to 5–10% of the lateral buds. The harvest date is later than that of ‘Chandler.’ The ‘Franquette’ nut size is small, and the kernel percentage is low at 47%.

### Dormancy Progression Using the Artificial Chill Methodology

#### Plant Material Collection and Experimental Design

In walnut, we can define three types of buds: (1) vegetative buds (which develop into leaves and shoots); (2) mixed buds (which first provide small shoots and then two female flowers); (3) catkin buds, which contain the primordium of a male inflorescence. The vegetative and mixed buds are externally similar and can be differentiated only through histological observations. For this reason, walnut buds are usually divided into vegetative and catkin buds in scientific literature. In this study, to simplify the nomenclature, vegetative buds refer to both vegetative and mixed buds, whereas male buds refer to catkins.

During the second fortnight of April (mid-autumn) of each year of the trial and at the start of field chill accumulation, determined from the minimum CU value (i.e., when chill negation is lower than chill accumulation), in the Utah model (Richardson et al., 1974), 1-year-old shoots were randomly collected from 10 ‘Chandler’ walnut trees (in 2012, 2013, and 2015) and 10 ‘Franquette’ trees (in 2013 and 2015).

Branches measuring 35–45 cm in length with a basal diameter of about 8–12 mm and a mean of 20 buds per twig, with an average of 80% vegetative buds and 20% male buds (catkins), were picked from trees in the field and transferred to the laboratory in controlled conditions. The trials were conducted in a completely randomized design following Dennis (2003) and Viti et al.’s (2003) criteria, with three replicates of four cuttings per treatment and cultivar. The treatments included the following factors: (a) chill hours (Bennet, 1949; Weinberger, 1950) in a cold chamber; (b) type of shoot (non-decapitated or decapitated); and (c) type of bud (vegetative or male bud).

#### Artificial Chill and Heat Treatments

Artificial chilling treatments were performed on non-decapitated and decapitated branches. This made it possible to simulate usual pruning conditions and to evaluate the paradormancy induced by correlative inhibition of the apical bud (Lang et al., 1987). After disinfection with fungicide [*N-(triclorometiltio) ciclohex-4-eno-1,2-dicarboximide*, 4,000 ppm (Captan™, Bayer CropScience, NC, United States)], bundles of 12 cuttings (four twigs and three repetitions per treatment) were wrapped in moistened cheesecloth and placed in plastic bags to prevent dehydration during low temperature treatments of 0, 300 (7.8), 600 (16.2), 750 (20.4), 900 (24.7), 1,050 (28.9), 1,200 (33.1), 1,350 (37.3), and 1,500 (41.6) h (Chill Portions) using a dark growth chamber (3 ± 0.5°C). This methodology was slightly modified from Aslamarz et al. (2009) and Vahdati et al. (2012). In our experimental conditions, using shoots with multiple buds, we chose 30% budbreak as the threshold for endodormancy release. Budbreak was defined as the “Cf” (balloon or green tip) stage in vegetative buds and “Cm” (male flower individualization) in male buds (Germain and Lespinasse, 2005). In order to facilitate the reading of units, we chose CH to represent the artificial chilling treatment at a constant temperature (3°C), even though this unit would be equivalent to the CU since temperatures within the 2.4–9.1°C range have a weighting factor of 1 in the Utah Model (Richardson et al., 1974).

After chilling, the shoots were placed in plastic containers with their basal tips in water and forced into a phytotron with a 12-h photoperiod, 40 μmol.m-2.s-1 light intensity, and 70-75% relative humidity at 20 ± 1°C for 8 weeks. Approximately 0.5–1 cm of the distal shoot section was cut off weekly to ensure that the vascular system remained functional (Citadin et al., 2003; Aslamarz et al., 2009). Vegetative and male budburst was recorded every 2–3 days.

The heat requirements were calculated as Growing Degree Hours (GDH°C) following the model proposed by Richardson et al. (1974), from the date on which the shoots were transferred to the growth chamber up until 30% of the buds reached the ‘Cf’ or ‘Cm’ phases. GDH (°C) was quantified for each cultivar, year, type of bud (vegetative or male), and shoot (non-decapitated or decapitated).

### Dormancy Progression Using the Field Chill Methodology

#### Plant Material Collection and Experimental Design

In addition to the forcing-chilling experiment described in Section “Dormancy Progression Using the Artificial Chill Methodology,” we also evaluated dormancy release upon chill accumulation in field conditions. One-year-old shoots were collected every 2–3 weeks, from mid-April (endodormancy) to mid-September (pre-bud burst), and immediately transferred to a forcing environment to stimulate budbreak. This experiment was carried out in ‘Chandler’ (2012–2016) and ‘Franquette’ (2013–2016) cultivars. In this trial, the sampling, evaluation, and analysis of the results followed the guidelines described in the previous section except in the case of chill accumulation, which was fulfilled in the trees grown in the experimental field.

The experiments were conducted under a completely randomized design following Dennis (2003) and Viti et al.’s (2003) criteria. Each treatment comprised a set of 12 excised shoots with three replications and four shoots per plot (an average of 60 vegetative buds and 20 male buds per treatment). For each walnut cultivar and year, the treatments included the following factors: (a) field chill date (calculated in CPs); (b) type of shoot (non-decapitated and decapitated); and (c) type of bud (vegetative or male).

#### Field Chill and Forced Heat Treatments

Four models were used to quantify CRs in walnut cultivars (reviewed in Luedeling, 2012): the Chilling Hours model (Bennet, 1949; Weinberger, 1950); the Utah model (Richardson et al., 1974); the Positive Utah Model (Linsley-Noakes et al., 1994); ad the Dynamic model (Fishman et al., 1987a,b; Erez et al., 1990). The chilling accumulation start date was considered from the minimum CU value in the Utah model (Richardson et al., 1974; Luedeling, 2012; Zhuang et al., 2016) for the three first models, whereas the Dynamic model starts automatically on the initial date of chilling accumulation (Fishman et al., 1987a,b; Erez et al., 1990).

Budbreak was evaluated following the same methodological protocol as that used for the artificial chilling treatments. When dormancy release was achieved (when 30% of the buds reached the stages mentioned above, ‘Cf’ and ‘Cm’), the corresponding GDH (°C) were recorded according to Richardson et al. (1974) and quantified for each cultivar, year, type of bud (vegetative or male) and type of shoot (non-decapitated and decapitated). Finally, to quantify chill and heat requirements, dormancy break was defined as the date on which a budbreak rate of 30% was obtained, after 30 days of forcing. This methodology was based on a widely used protocol in fruit trees (Ruiz et al., 2007; Alburquerque et al., 2008; Campoy et al., 2010, 2019), but it was adapted to the slower walnut bud growth. We considered dormancy release to have occurred after 30 days under forcing conditions as opposed to the 10 days used by the aforementioned authors. It should be noted that we chose the budbreak percentage of 30% rather than the 50% of Aslamarz et al. (2009) and Vahdati et al. (2012) in order to avoid further increasing the number of forced days. Given the availability of plant material and the impossibility of sampling at a frequency of two-three times per week (Ruiz et al., 2007), an interpolation of the GDH and CP accumulation was carried out to estimate the CR and HR. CP was used to represent the dormancy progression as it is a more robust unit (Fishman et al., 1987a,b).

### Meteorological Data

The meteorological data were recorded from a Davis Instruments ^®^ wireless automatic meteorological station with ISO 9001 MB3LR certification (the Vantage Pro2 model, Davis Instruments Corporation, CA, United States), installed at EEAVI bordering the walnut collection, with data resolution every 15 min.

### Histological Analysis

During the 2015 and 2016 seasons, from July to October (end of endodormancy to pre-bud burst), excised samples of the female terminal and lateral buds and male buds of ‘Chandler’ and ‘Franquette’ cultivars were fixed in an FAA solution (Ruzin, 1999) for at least 24 h, dehydrated through an ascending series of terbutyl alcohol and embedded in paraffin (Sigma-Aldrich, United States; Jensen and Towne, 2015). Vertical and cross-sections with a thickness of 8–10 μm were cut with a rotary microtome (Minot, Jung, Germany), transferred to glass slides, dried overnight, and stained with safranin-fast-green (Ruzin, 1999). Photographs were taken on a Nikon Labophot-2 microscope (Nikon Instruments Inc., Melville, United States). Five replicates were used for each sampling, which means that we prepared, observed, and interpreted a total of 400 serial cuts of the material extracted from the field, including 250 cuts in the 2015 season and, after selecting key sampling dates based on the 2015 results, 150 cuts in the 2016 season.

### Quantification of Soluble Sugars

Throughout the 2015 season, on the same dates as those indicated for microscopy preparations, excised ‘Chandler’ female and male buds were ground to a powder in a mortar, frozen at –35°C, and then lyophilized at –40°C and stored in a desiccator prior to analysis. Afterward, 10 buds were used for each biological replicate, whereas for each sample date we measured two replicates.

Sample preparation followed the Association of Official Agricultural Chemists standards (Aoac 925.05., 1990).

The soluble sugar contents were measured using high-performance liquid chromatography (HPLC), coupled to a UV detector, following the method used by Martínez Ruiz (2005), with some modifications. We used an Alliance e2695 chromatographic system (Waters, United States) equipped with a Rezex ROA-Organic Acid column (Phenomenex, United States) of 300 × 7.8 mm, a 10 μl injection loop, and a 2414 Refractive Index Detector (Waters, United States). As a mobile phase, a 5 10-3N H_2_SO_4_ solution was used with a flow rate of.3 ml/min, and the temperature of the column was kept at 30°C. For data processing, the Empower 2 (Shimadzu, Japan) program was used. The following carbohydrates were used as standards: sucrose, glucose, and fructose (Sigma-Aldrich, United States, purity ≥ 99%).

Quantification was carried out by comparison of areas through the external standard method, using solutions of the different soluble sugars in concentrations of 0.1–10 mg/ml as a reference. The typical retention times of the analyzed sugars, as well as the calibration curves, were obtained for each sugar standard. Sugar extract portions (5 ml) of the samples were taken and lyophilized to eliminate traces of water in the samples and then rehydrated using 5 ml of mobile phase (H_2_SO_4_ 5 10-3N) and filtered through a 0.45-μm Millipore filter to collect approximately 2 ml of solution. Injections were done in duplicate. The analyses were carried out at the PLAPIQUI (CONICET) Lab in Bahía Blanca, Argentina.

### Statistical Analysis

Artificial-chill trials were analyzed using an ANOVA test in the InfoStat ^®^ program (National University of Córdoba, Argentine) (Di Renzo et al., 2008), whereas field-chill trials were analyzed using Kruskal–Wallis tests in the R package (R Core Team, 2020, R Foundation for Statistical Computing, Vienna, Austria). For both trials, we evaluated the differences in GDH. For each walnut cultivar and year, the procedures included the following variables: (a) forced chill treatments (0–1,500 Chill Hours) or field chill dates (calculated in CPs); (b) type of shoot (non-decapitated and decapitated); (c) type of bud (vegetative or male). The significance threshold was established at 0.05.

## Results

### Dormancy Progression Using the Artificial Chill Methodology

**FIGURE 1:**
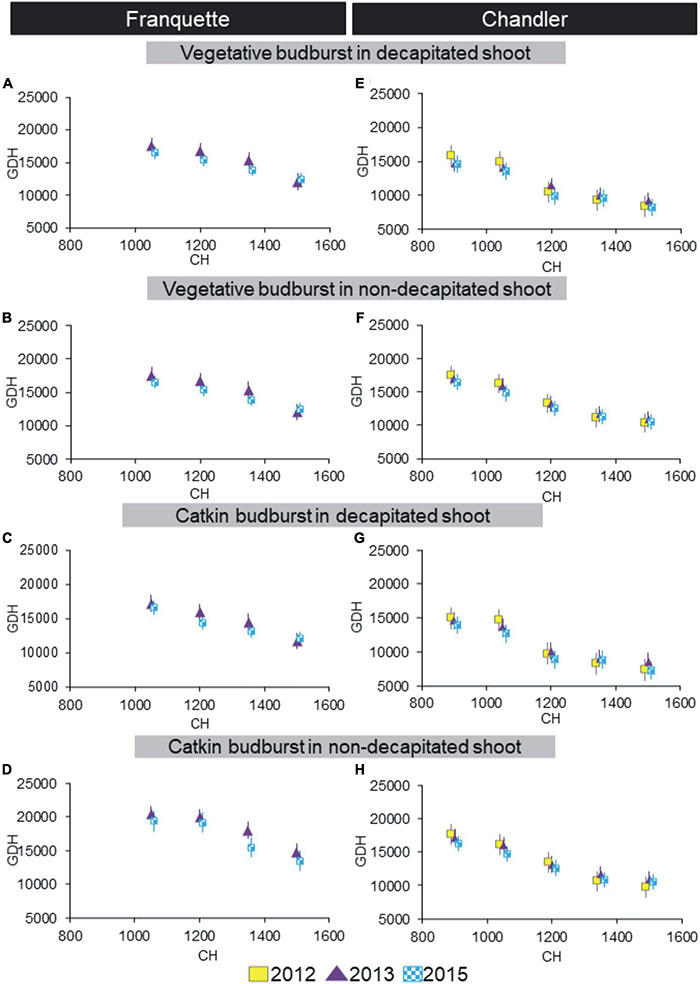
Dormancy progression in ‘Franquette’ (years 2013 and 2015, **A–D**) and ‘Chandler’ (years 2012, 2013, and 2015, **E–H**) walnut cultivars using the artificial chill methodology. Time to budbreak is expressed as heat requirements, using Growing Degree Hours (GDH°C) (Richardson et al., 1974), whereas artificial chill treatments are expressed in chill hours (CH) (Bennet, 1949; Weinberger, 1950). Results are shown for vegetative and male (catkin) buds in decapitated and non-decapitated shoots. Vertical bars represent standard errors of the mean values.

Our experimental design allowed us to evaluate dormancy progression using artificial chill according to the bud type (vegetative or male) and pruning state (decapitated or non-decapitated shoots) for both the ‘Franquette’ and ‘Chandler’ cultivars (Figure 1). The higher the amount of artificial chill applied, the lower the heat requirements (expressed as GDH). The artificial treatments of 0, 300, 600, 750 CH were not able to induce bud break for any cultivar, nor was 900 CH in ‘Franquette’ (therefore, data not shown in Figure 1). In both treatments, in decapitated and non-decapitated shoots, the greatest decreases in GDH were observed when artificial chill increased from 1,050 to 1,200 CH in ‘Chandler’ and from 1,350 to 1,500 CH in ‘Franquette.’ For a given value of heat requirements to budburst, ‘Chandler’ needed fewer CH than ‘Franquette.’ In relation to this, the decrease in GDH in ‘Chandler’ from 1,350 CH on was minimal compared to the decrease observed in previous artificial chill values. This shows that additional chilling from the 1,350 thresholds did not affect GDH. This trend was not found in ‘Franquette.’ Decapitating the shoots significantly reduced the necessary CH (CR) for a given heat requirement value in both cultivars.

### Dormancy Progression Using the Field Chill Methodology

**FIGURE 2:**
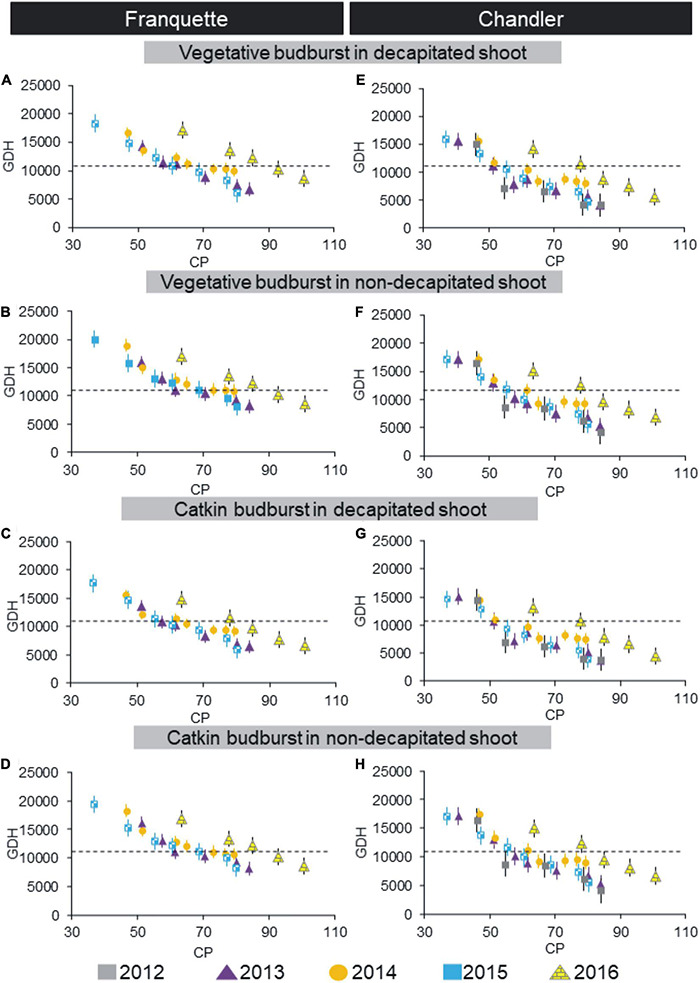
Dormancy progression in ‘Franquette’ (years 2013, 2014, 2015, and 2016, **A–D**) and ‘Chandler’ (years 2012, 2013, 2014, 2015, and 2016, **E–H**) walnut cultivars using the field chilling methodology. Time to budbreak is expressed as heat requirements, using Growing Degree Hours (GDH°C) (Richardson et al., 1974), whereas field chill treatments are expressed in chill portions (CP) (Fishman et al., 1987a,b; Erez et al., 1990). Results are shown for vegetative and male (catkin) buds in decapitated and non-decapitated shoots. Dotted lines represent the GDH threshold for dormancy release. Vertical bars represent standard errors of the mean values.

Using the field chill methodology, we also evaluated dormancy progression according to the bud type and pruning state for both cultivars (Figure 2). We found significant differences in GDH between cultivars (lower in ‘Chandler’ than in ‘Franquette’), shoot type (lower in decapitated shoots than in non-decapitated), and bud type (male earlier than vegetative). Significant differences were also observed among GDH in different years of the trials. Year-by-year variability increased with the abnormal year 2016, which showed much higher variability than previous years. We also calculated the dormancy release dates (30% budburst after 30 days, corresponding to 11,160 GDH) (Table 1). The dormancy release dates ranged from July 12 to July 31 for ‘Chandler’ and from July 28 to August 12 for ‘Franquette’ (Table 1). ‘Chandler’ required an average of 10.3 fields CPs less than ‘Franquette’ for dormancy breaking.

**TABLE 1:** Chilling requirements for dormancy release of vegetative buds estimated using walnut non-decapitated cuttings (Field Chill) from ‘Chandler’ and ‘Franquette’ cultivars.

Cultivar	Dormancy breaking	Chill estimation models			
		CH	CU	Positive CU	Portions
Chandler	July-12-2012	771	920	1082	51.3
	July-22-2013	759	880	996	53.3
	July-12-2014	641	1015	1166	58.1
	July-19-2015	666	762	1118	55.4
	July-31-2016	968	1536	1556	78.7
	Mean	761.0	1022.6	1183.6	59.7
	*SD*	128.8	301.0	217.3	12.8
	C.V.	16.9	29.4	18.4	21.4
Franquette	August-03-2013	878	1068	1188	62.4
	July-28-2014	830	1212	1375	68.6
	August-05-2015	782	898	1288	63.2
	August-12-2016	1072	1679	1726	85.9
	Mean	890.5	1214.3	1394.3	70.0
	*SD*	127.2	335.4	234.0	10.9
	C.V.	14.3	27.6	16.8	15.6

*CH, chill hours model, the number of hours when the temperature is between 0 and 7.2°C; CU, chill units, Utah model; PosCU, positive chill units, a modified version of the Utah model; CP, chill portions, dynamic model; SD, standard error. CV, coefficient of variation.*

The heat requirements of the cultivars (GDH) were reduced by continuous chill accumulation throughout the dormancy period studied.

As in the artificial chilling experiment, we found that decapitated shoots needed the equivalent of 3–5 days less GDH than non-decapitated shoots. Similarly, we found a decrease in GDH of up to 2 days in male vegetative buds with respect to vegetative buds. As for the cultivars, ‘Franquette’ needed an average of 2,163 GDH°C (equivalent to 6 days) more than ‘Chandler.’

The dormancy breaking dates and CR according to field chill accumulation are summarized in Table 1. High between-year variability was found in the dormancy breaking date and CR, with coefficients of variation of CP of 21.4 and 15.6 for ‘Chandler’ and ‘Franquette,’ respectively.

**FIGURE 3:**
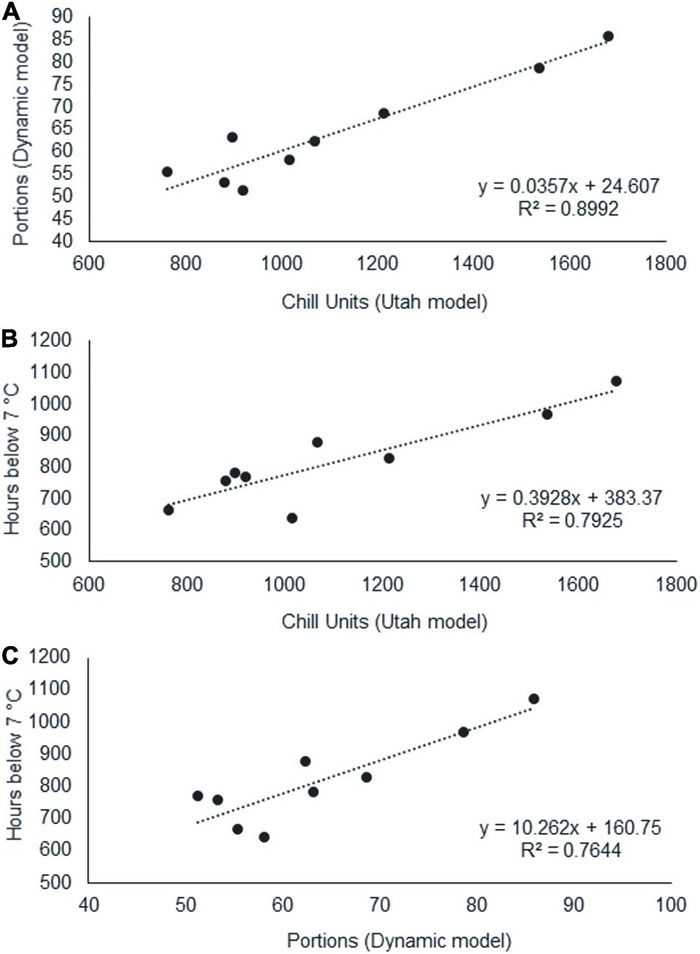
Linear relationships between chilling requirements for dormancy breaking calculated the Utah model (Chill Units, CU) (Richardson et al., 1974), Dynamic model (Chill Portions, CP) (Fishman et al., 1987a,b; Erez et al., 1990), and Chill Hours model (Hours below 7°C, CH). CP-CU **(A)**, CH-CU **(B)**, and CH-CP **(C)**.

Once we calculated the CR using different methods, we evaluated the prediction of the CR calculated with the different chill models evaluated (Figure 3). We found a high coefficient of determination (*R*^2^: 0.9) between CU and CP and moderate ones (*R*^2^: 0.79 and 0.76) between CH and CU and CH and CP, respectively.

#### Anatomical Changes Were Observed in Buds During the Endo-Ecodormancy Progression

**FIGURE 4:**
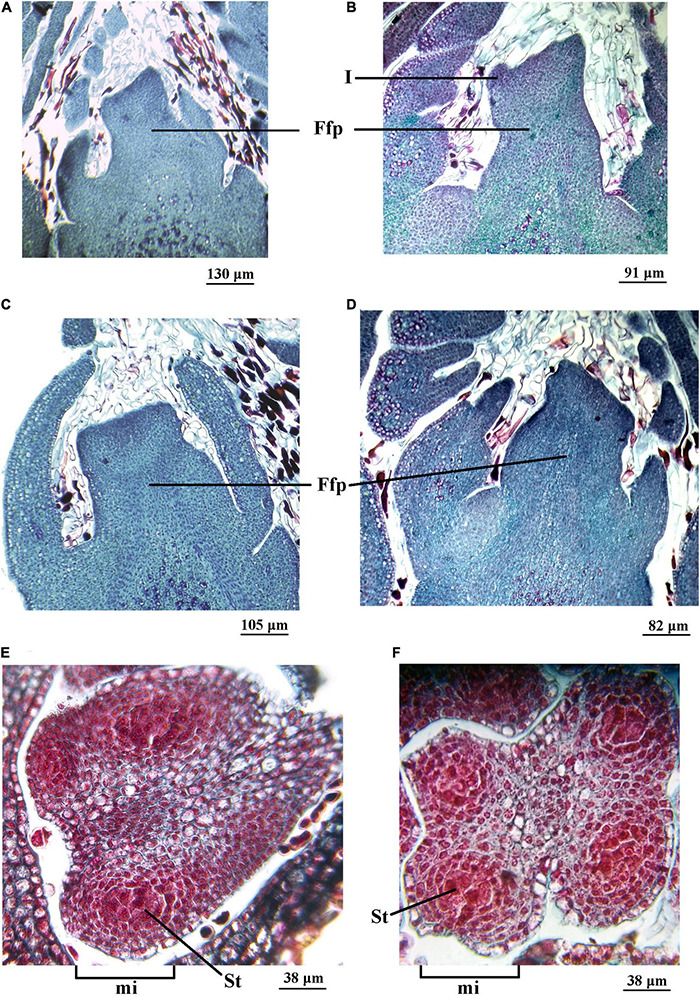
Female (vegetative-mixed) and male (catkin) reproductive structures of two *Juglans regia* L cultivars (cv.) sampled on July 20 and 31 of 2015, respectively. Longitudinal section of the female floral primordium (Ffp) of an apical bud of ‘Chandler cv. **(A)** and ‘Franquette’ cv. **(B)**. Longitudinal section of the Ffp of a lateral bud of ‘Chandler’ cv. **(C)** and ‘Franquette’ cv. **(D)**. The center of each microsporangium (mi) contained sporogenous tissue (St) in ‘Chandler’ cv. **(E)** and ‘Franquette’ cv. **(F)**. I, involucre.

On July 20, 2015, in the ‘Chandler’ cultivar, vertical sections of vegetative-mixed (female) terminal buds showed a flattened apex with an incipient ring of tissue at the edge, showing the initial development of the involucre (Figure 4A). The apex of the lateral bud was completely flattened in a less developed stage (Figure 4C). Inside the anthers, the four microsporangia were observed with the sporogenous tissue in formation (Figure 4E).

In the ‘Franquette’ cultivar, on July 31, 2015, 11 days later than the sampling for the Californian ‘Chandler,’ vertical sections of female terminal buds showed that the meristematic tissue that generates the involucre was slightly more developed than in ‘Chandler,’ with two protrusions that indicated the start of the perianth as a second ring of tissue (Figure 4B). The development of the lateral bud (Figure 4D) and that of the anthers (Figure 4F) was similar to that observed in ‘Chandler’ on July 20, 2015. The similarity in anatomical structures agrees with the similar difference between the sampling dates (11 days) and the dormancy release dates in both cultivars (17 days, ‘Chandler’: 19 July; ‘Franquette’: 5 August; see Table 1).

**FIGURE 5:**
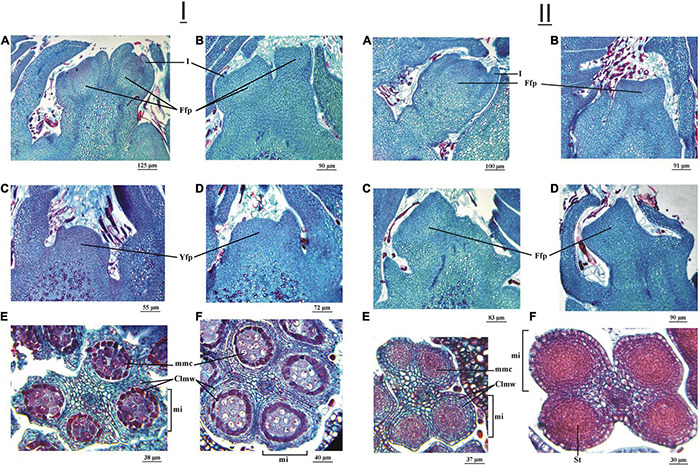
**(I)** Female and male reproductive structures of two *J. regia* L. cultivars (cv.), ‘Chandler’ cv. and ‘Franquette’ cv., sampled on July 11, 2016. Longitudinal section of a young floral primordium (Yfp) of an apical bud of ‘Chandler cv. **(A)** and ‘Franquette’ cv. **(B)**. Longitudinal section of a Yfp of a lateral bud of ‘Chandler’ cv. **(C)** and ‘Franquette’ cv. **(D)**. The microsporangia (mi) of ‘Chandler’ cv. **(E)** and ‘Franquette’ cv. **(F)** with microspore mother cells (mmc). Clmw, cell layers of the microsporangium wall. I, involucre; S, sepal. **(II)** Female and male reproductive structures of two *J. regia* L. cultivars (cv.), ‘Chandler’ cv. and ‘Franquette’ cv., sampled on August 24, 2016. Longitudinal section of a female floral primordium (Ffp) of an apical bud of ‘Chandler’ cv. **(A)** and ‘Franquette’ cv. **(B)**. Longitudinal section of a Ffp of a lateral bud of ‘Chandler’ cv. **(C)** and ‘Franquette’ cv. **(D)**. ‘Chandler’ cv. **(E)** microsporangia (mi) with microspore mother cells (mmc). ‘Franquette’ cv. **(F)** microsporangia with the sporogenous tissue (St). Clmw, cell layers of the mi wall.

In the cultivar ‘Chandler,’ on July 11, 2016, the longitudinal section of the apical bud showed the flower primordia with evident development of the involucral ring (Figure 5IA). In ‘Franquette,’ on the other hand, as expected, such development was only incipient (Figure 5IB). The longitudinal sections of the axillary bud of both cultivars showed the apical meristems of the primordia in a typically convex juvenile phase, that is, dome-shaped (Figures 5IC,D). The male buds of both cultivars had anthers in whose microsporangia the stem cells of the microspores could be observed, and there were no differences between cultivars in the degree of development of these structures (Figures 5IE,F). In this year, the dormancy release dates of the ‘Chandler’ and ‘Franquette’ cultivars were July 31 and August 12, respectively (Table 1).

**FIGURE 6:**
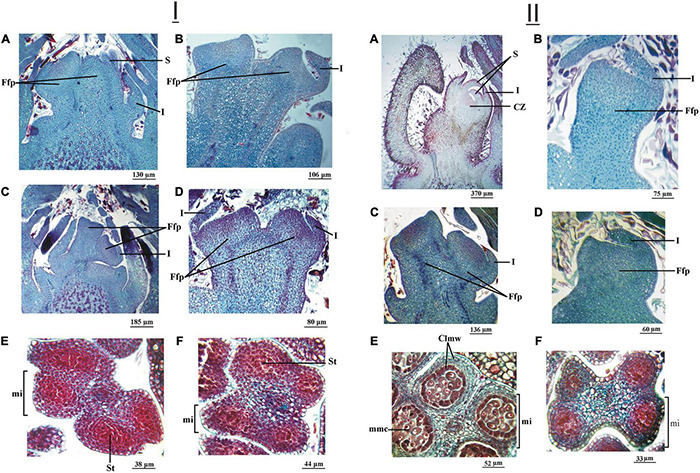
**(I)** Female (vegetative-mixed) and male (catkin) reproductive structures of two *J. regia* L. cultivars (cv.) sampled on August 19, 2015. Longitudinal section of the female floral primordium (Ffp) of an apical bud of ‘Chandler’ cv. **(A)** and ‘Franquette’ cv. **(B)**. Longitudinal section of the Ffp of a lateral bud of ‘Chandler’ cv. **(C)** and ‘Franquette’ cv. **(D)**. The center of each microsporangium (mi) contained sporogenous tissue (St). In ‘Chandler’ cv. **(E)** and ‘Franquette’ cv. **(F).** I, involucre; S, sepal. **(II)** Female and male reproductive structures of two of *J. regia* L. cultivars (cv.) sampled on September 30, 2015. Longitudinal section of the female floral primordium (Ffp) of an apical bud of ‘Chandler’ cv. **(A)** and ‘Franquette’ cv. **(B)**. Longitudinal section of the Ffp of a lateral bud of ‘Chandler’ cv. **(C)** and ‘Franquette’ cv. **(D)**. The microsporangia (mi) of ‘Chandler’ cv. **(E)** contained microspore mother cells (mmc). ‘Franquette’ cv. **(F)** microsporangia still presented the sporogenous tissue (st). Clmw, cell layers of the microsporangium wall; CZ, carpelar zone; I, involucre; S, sepal.

In excised samples of female terminal buds on August 19, 2015, observation date (30 and 14 days after dormancy breaking in ‘Chandler’ and ‘Franquette,’ respectively; refer to Table 1), ‘Chandler’ showed the most developed involucre and the beginning of perianth (Figure 6IA), while ‘Franquette’ showed less development (Figure 6IB). On this date, the lateral bud and anther development in ‘Franquette’ and ‘Chandler’ were similar. The apex of the lateral buds showed involucre development (Figures 6IC,D), while in male buds, the wall of the microsporangia showed the first periclinal divisions destined to generate the four-cell layers located beneath the external epidermis (Figures 6IE,F).

In the August 24, 2016 observations, involucre development was noted in the female apical bud of the cultivar ‘Chandler’ (Figure 5IIA). This was not the case in ‘Franquette,’ in which the meristematic apex appeared flattened with just the initiation of the involucre ring (Figure 5IIB). The longitudinal sections of the axillary buds in both cultivars showed the flower primordia with their apices already flattened (Figures 5IIC,D). In ‘Chandler,’ the anthers presented microsporangia with stem cells of the microspores (Figure 5IIE). In the cultivar ‘Franquette,’ sporogenic tissue was observed, with no differentiation of the microspore stem cells in the later genotype (Figure 5IIF).

On the September 30, 2015, sampling date, the apex of the apical female bud of ‘Chandler’ showed the involucre, and the four sepals already covered the apical meristem. The carpels formed a ring of tissue that left a small depression in the center. The lateral bud showed similar involucre development to the apical bud on the previous sampling date (Figures 6IIA–C). In the anther, all the cellular layers of the microsporangium wall were differentiated, and the microspore stem cells in the center could be observed (Figure 6IIE). In ‘Franquette,’ the development of the floral primordium in the apical and lateral buds was similar to that of the previous sampling date (Figures 6IIB–D). In the microsporangia wall, the layers that would generate the four-cell strata located below the external epidermis were evident, and the differentiation of the future stem cells from the microspores was more evident (Figure 6IIF).

**FIGURE 7:**
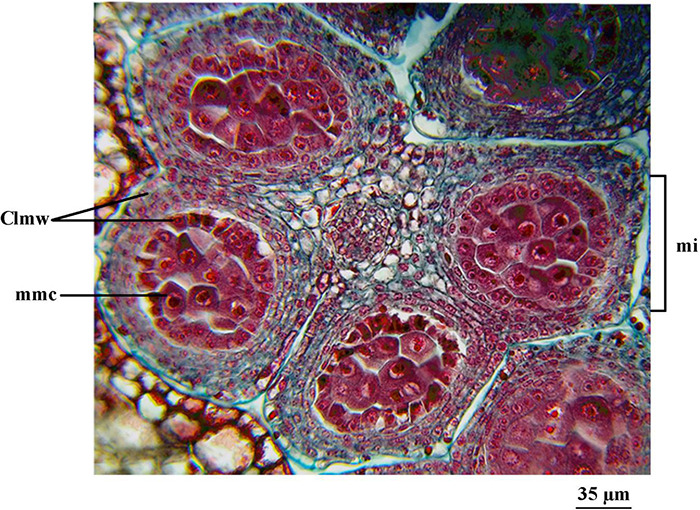
Sampling of male buds (catkins) from *J. regia* L. cv. ‘Franquette’ corresponding to October 19, 2015. Cross-section of an anther of a cv. ‘Franquette’ male bud. cmm, microspore stem cell. E, cell strata. M, microsporangium.

The ‘Franquette’ male buds (Figure 7) did not reach a development equivalent to that observed in ‘Chandler’ on September 30, 2015, until October 19, 2015, sampling date. Both dates were prior to the respective cultivar bud burst stages.

**FIGURE 8:**
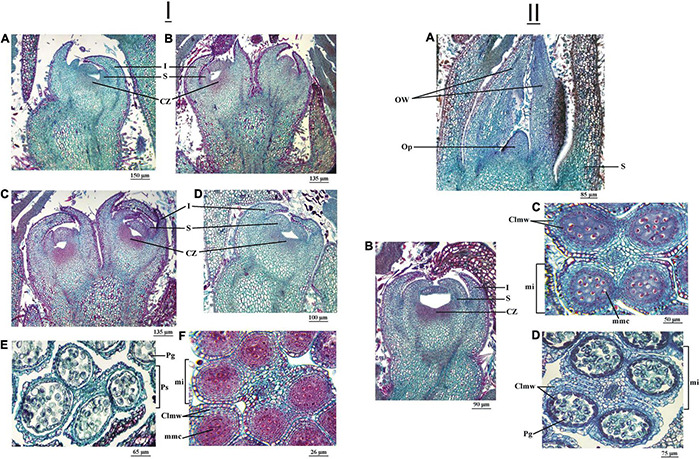
**(I)** Female and male reproductive structures of two *J. regia* L. cultivars (cv.), ‘Chandler’ cv. and ‘Franquette’ cv., sampled on September 23, 2016. Longitudinal section of a female floral primordium (Ffp) of an apical bud of ‘Chandler’ cv. **(A)** and ‘Franquette’ cv. **(B)**. Longitudinal section of an Ffp of a lateral bud of ‘Chandler’ cv. **(C)** and ‘Franquette’ cv. **(D)**. Pollen sacs (Ps) of ‘Chandler’ cv. **(E)** with immature pollen grains (Pg). Microsporangium of ‘Franquette’ cv. **(F)** with microspore mother cells (mmc). Clmw, cell layers of the mi wall; CZ, carpelar zone; I, involucre; S, sepal. **(II)** Female and male reproductive structures of a *J. regia* L. cultivar, ‘Franquette’ cv., sampled on October 5 **(A–C)** and 15 **(D)** of 2016. Longitudinal section of a female floral primordium (Ffp) of an apical **(A)** and a lateral bud **(B)** in ‘Franquette’ cv. **(C)** Microsporangium (mi) with microspore mother cells (mmc) and ten days later **(D)** with mature pollen grains (Pg). Clmw, cell layers of the mi wall; CZ, carpelar zone; I, involucre; OW, ovary wall; Op, ovule primordium; S, sepal.

In the September 23, 2016 observations, the development reached by the flower primordia of the apical buds was similar in both cultivars. The development of the involucre and the sepals was very evident and, in turn, the carpelar leaf had begun to differentiate (Figures 8IA,B). In the floral primordium of the lateral bud of the cultivar ‘Chandler,’ a marked depression was observed in the carpelar area where the ovary would later differentiate (Figure 8IC). In the cultivar ‘Franquette,’ this development was incipient (Figure 8ID).

In ‘Chandler,’ the anther microsporangia showed immature pollen grains (Figure 8IE), whereas, in ‘Franquette,’ the microsporangia contained cells at the microspore stem cell stage (Figure 8IF).

On October 5, 2016, only ‘Franquette’ was sampled since its reproductive structures develop later than those of ‘Chandler.’ In fact, ‘Chandler’ was already in the bud burst stage in the field on this sampling date. In the flower primordia of the apical buds in ‘Franquette,’ advanced development of the involucre, sepals, and carpelar zone was observed (Figure 8IIA). The placenta appeared as a central column, free from the carpel wall (Figure 8IIB). In the floral primordium of the lateral bud, a depression could already be observed in the carpelar area where the ovary would later differentiate (Figure 8IIC). In the male buds, the anthers presented microsporangia with microspore stem cells (Figure 8IID).

**FIGURE 9:**
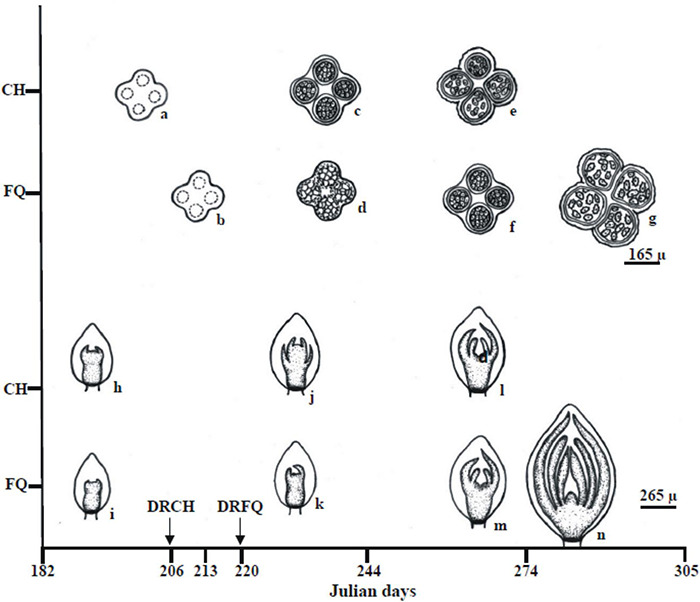
Graphical summary of the mean (2015–2016) developmental state of male (a–g; anther cross-sections) and female (h–n, apical bud longitudinal sections) reproductive structures of the ‘Chandler’ (CH) and ‘Franquette’ (FQ) *J. regia* L. cultivars. Microsporangia with sporogenous tissue (a,b,d), microspore mother cells (c,f), and immature (e) and mature (g) pollen grains. Female floral primordiums (Ffp) with incipient (i) and more advanced involucre development (h). Ffp with involucral ring fully developed (j,k). Sepal primordiums have appeared in Chandler (j) only. Carpelar zone differentiation became evident as a depression in the center of the apical meristem (l,m). The ovary and an ovule primordium were present (n).

Figure 9 shows a summary outline of the male and female reproductive structure development of the ‘Chandler’ and ‘Franquette’ *J. regia* L cultivars.

#### Quantification of Soluble Sugars, Sucrose, and Hexoses. High-Performance Liquid Chromatography Analysis

**FIGURE 10:**
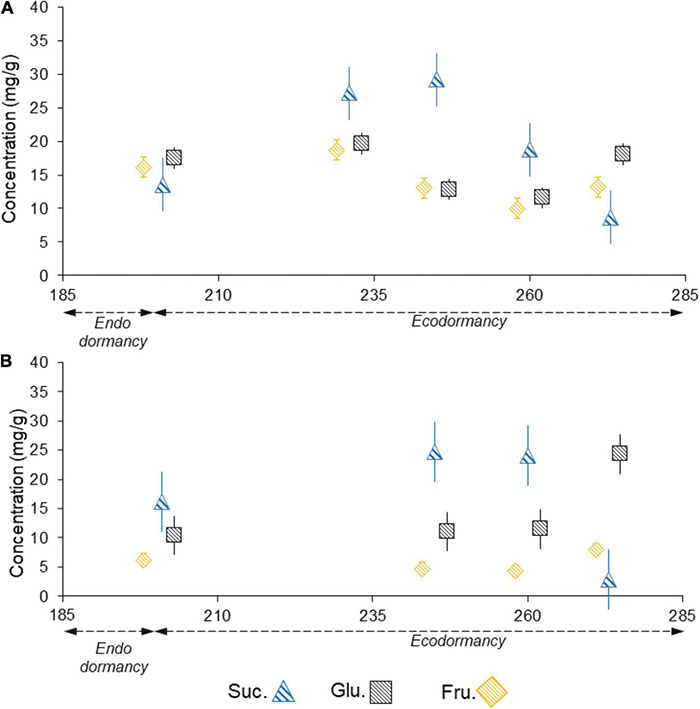
Sucrose, glucose, and fructose content in vegetative -mixed- buds **(A)** and male buds **(B)** of the walnut cultivar ‘Chandler’ sampled from field shoots from July to September 2015.

The anatomical analyses were complemented with the study of soluble sugars for one season in ‘Chandler,’ from July to September 2015. The soluble sugar progression showed a different pattern in female (vegetative mixed) buds (Figure 10A) and male buds (Figure 10B). In female buds, sucrose had already started decreasing by the beginning of September, whereas in male buds, sucrose increased until the middle of September and then decreased. The fructose and glucose levels were within the same range in female buds, whereas in male buds, glucose levels were higher than fructose throughout the period studied. In both types of buds, the decrease in sucrose preceded an increase in the fructose and glucose levels, with the latter prevailing.

## Discussion

The artificial chill accumulation methodology has been widely used in various fruit species including walnut (Citadin et al., 2001; Gariglio et al., 2006; Afshari et al., 2009; Aslamarz et al., 2009; Charrier et al., 2011; Vahdati et al., 2012; Li et al., 2016). Nonetheless, Dennis (2003) noted that, with this approach, the results are likely to be questionable outside of a controlled environment. In addition, Luedeling et al. (2009c) observed lower field CRs than artificial CRs in the walnut cultivar ‘Scharsh Franquette.’ These results agree with our own in this study, in which the artificial CRs were higher and showed lower between-year variability than field CRs (Figures 1, 2).

The field chill accumulation approach has also been broadly used in fruit tree research (Balandier et al., 1993; Ruiz et al., 2007, 2018; Guo et al., 2013; Zhuang et al., 2016; Hassankhah et al., 2017). Unlike artificial chill, field chill allows for the interannual variability of the air temperature during the subsequent autumn-winter months.

Several models have been suggested for calculating winter chill (Luedeling et al., 2009a,b,c). The Dynamic model has achieved widespread consideration in recent years due to its robust design based on more experimental data (Fishman et al., 1987a,b) and its satisfactory performance in different environments (Luedeling et al., 2009b,c; Darbyshire et al., 2011; Luedeling and Brown, 2011; Zhang and Taylor, 2011; Miranda et al., 2013).

In our results, the regression between the CU/CP models achieved a coefficient of determination of 0.9. Previous studies, in different fruit species, have also reported this high value between both parameters (Erez et al., 1990; Ruiz et al., 2007; Campoy et al., 2012). Consequently, for northern Patagonian field conditions, both models could be potentially used to estimate winter chill needs in walnut cultivars, although we would recommend the use of CP, as it is widely accepted in agricultural research (Luedeling, 2012; Campoy et al., 2013; Benmoussa et al., 2017a; Egea et al., 2021) and agricultural planning and management (Pérez et al., 2008; Luedeling and Brown, 2011). However, a lower coefficient of determination has been found between the CU or CP and CH models (*R*^2^: 0.79 and 0.76, respectively). This indicates that the Chill Hours Model, widely used by growers due to its simplicity and easy calculation, provides an indicative value of winter chill conditions for breaking dormancy in walnut, but its reliability is lower than that of the two methodologies mentioned above.

On this basis, the CRs for dormancy release of both walnut cultivars studied are satisfied in northern Patagonia (Martín, 2009; Musi Saluj, 2019). The conversion factor CH – CP is a good driver for the extrapolation of results among different variables and is site-specific (Luedeling and Brown, 2011). In this study, we present the first CH – CP ratio (12.8) for northern Patagonian conditions calculated in dormancy release experiments.

In artificial chill trials, the heat requirements for budburst showed an average decreasing rate of 6,000–7,000 GDH°C, with overexposure to chilling temperatures beyond 900 CH (70 CP) (‘Chandler’s’ dormancy break) and 1,050 CH (82 CP) (‘Franquette’s dormancy break) to 1,500 CH. In field chill trials, once the cultivars fulfilled the CP for dormancy break, chill overexposure resulted in even more decreases in GDH°C. These results agree with studies carried out in various fruit trees species (Couvillon and Erez, 1985; Gariglio et al., 2006; Okie and Blackburn, 2011; Campoy et al., 2012; Hussain et al., 2015; Li et al., 2016), as well as in walnut (Aslamarz et al., 2009; Hassankhah et al., 2017). Our results also showed ‘in parallel’ rather than ‘sequential’ chill-heat accumulation, in agreement with Okie and Blackburn (2011), and a negative correlation between chill and heat requirements, as shown for a wide number of temperate tree species (Wang et al., 2020).

In field chill trials, regardless of the cultivar or treatment, within-year variability for CP/HR was lower than between-year variability, especially due to the effect of the abnormal year 2016 (Figure 2). The 2016 season had atypical winter and spring thermal conditions (Musi Saluj, 2019), which translated into an outlier distribution of dormancy progression compared to previous years. The influence of environmental factors can thus induce variability in dormancy progression, probably associated with year-to-year atmospheric and soil conditions, such as temperature (Welling, 2003), nutrition (Almond and Young, 1990), sunlight, rain, or water stress (Buchanan et al., 1977). The notable variability of dormancy progression and CR in this study highlights the need for multi-year studies in order to capture the variability of CR of cultivars in a region, as previous studies have shown in walnut (Aslamarz et al., 2009; Vahdati et al., 2012).

Endodormancy release is driven by the fulfillment of CRs. In this study, the heat requirements were the main driver of ecodormancy progression until bud burst, and the flowering processes, insomuch as the winter chill needs of fruit trees in cold temperate areas are usually overfulfilled. These results agree with the findings of Alonso et al. (2005) in almond trees growing in the cold temperate conditions of Zaragoza in northeastern Spain. Conversely, in the Mediterranean climatic conditions of Murcia, in southeastern Spain, Alburquerque et al. (2008) concluded that CRs had the greatest influence on flowering dates in sweet cherry cultivars. In walnut, Aslamarz et al. (2009) observed that flowering date in late-flowering walnut cultivars in warm areas and milder winters (Karaj, Iran) was mainly determined by the winter chill needs, although late-flowering cultivars showed higher heat requirements than early-flowering cultivars. Therefore, in northern Patagonia, the accumulation of heat in early spring is the main driver for the differences in bud burst and flowering dates among cultivars.

Regarding the effect of paradormancy on dormancy progression, we found that, regardless of the experimental protocol used (artificial/field chill accumulation), bud type or cultivar, decapitated shoots showed earlier budburst dates than cuttings that retained apical buds. This is a direct signal of paradormancy or correlative inhibition processes in the cultivars of the species studied and contrasts with Cline and Deppong’s (1999) observations in other species. Moreover, pruning the apical bud advanced the bud burst dates of the lateral buds in both walnut cultivars studied. These results are consistent with studies in apricot in which decapitated shoots showed the highest sprouting rates (Campoy et al., 2011a). This is also consistent with the stimulating effect of cutting in overcoming dormancy and with the fact that lateral buds have a shallower endodormancy (they need fewer chill hours) than terminal buds during the dormancy period (Champagnat, 1983; Rageau and Mauget, 1987; Crabbé and Barnola, 1996). Although some studies in peach and walnut buds (Scalabrelli and Couvillon, 1986; Aslamarz et al., 2009) have shown that the terminal buds require less chill accumulation than the lateral ones, in these studies the experimental shoots contained a single bud (lateral or terminal). Vitasse and Basler (2014) proved that the results obtained using cuttings with multiple buds are physiologically closer to adult tree performance and achieve phenological responses similar to those of the donor trees. This effect of pruning upon dormancy release is of key importance in walnut management in order to minimize the risk of spring frosts in northern Patagonia. Concurrently, late frost mostly affects male walnut buds in this region. ‘Chandler’ and ‘Franquette,’ the most cultivated genotypes in the region, are both protandrous, i.e., bud burst occurs earlier in male buds than in female buds. This hinders self-pollination in the event of late frosts and severely affects productivity. In the first study in northern Patagonian conditions, del Barrio and Martín (2011) found a higher recurrent risk of damage due to late frosts in ‘Chandler’ (33%) than in ‘Franquette’ (17.5%). These values suggest the need to develop high-chill/heat walnut cultivars for cold-temperate regions to reduce the risk of spring frost damage. This goal could be met by exploring the genetic diversity of walnut (Bernard et al., 2018) through breeding programs (Artyukhova, 2021).

The microscopy observations carried out in buds confirm a delay of 11 days in the development of bud anatomical structures in ‘Franquette’ with respect to ‘Chandler.’ These findings are in line with the higher CRs for dormancy release and, fundamentally, the higher heat requirements for spring bud burst of the French genotype compared to the Californian one (Luedeling et al., 2009c).

The processes involved in the differentiation sequences of both the female and male buds described above vary according to the cultivar and the climatic conditions (Zhang et al., 1995; Polito and Pinney, 1997). According to Gao et al. (2014) this sequence lasts about 1 year, integrating two growing seasons.

In our July 2015 and 2016 microscopic observations, with the female buds in full endodormancy or prior to dormancy release, we observed meristematic structures in the tissues of both cultivars with a large nucleus whose primary function is dividing, so it does not initially need cytoplasm or other types of developed cellular organelles. These meristematic tissues are prepared to divide and to start a process of differentiation or specialization toward more complex cellular vascular and reserve structures (Taiz and Zeiger, 2002). In the August 2015 and 2016 bud observations, 15–30 days after endodormancy release and during the eco-dormancy stage, there were vacuolated cells, which reflects the opening of the xylemic conduction channels allowing for water transport (Figures 5II, 6I). This implied the progressive loss of cold resistance in buds and a progressive increase in the risk of late frost damage (Charrier et al., 2011).

In the latest observations, in September-October of 2015 and 2016, just in the pre-bud burst stage, the density of nuclei in the apical and lateral female buds drops substantially and a large vacuole can be seen in the cells (Figures 6II, 8I,II). This agrees with Charrier et al. (2013), who determined that the water content decreases significantly in walnut buds in the autumn–winter and then increases from early spring until bud burst. But they indicate that this rehydration diverges strongly according to the relative position of the buds on the branch and that the water content is significantly higher in the apical buds than in the lateral ones. The roots begin to absorb water again and the aerial plant tissues are rehydrated (Ewers et al., 2001; Turcotte et al., 2009). Améglio et al. (2002) showed that hydration in walnut trees occurs when soil temperatures at a depth of 50 cm rise above 8°C.

On the September 30, 2015 sampling date, the involucre and the four sepals of the ‘Chandler’ apical female buds already covered the apical meristem. The lateral buds, on the other hand, showed less evolution—their development was similar to that of the apical buds on the previous sampling date in August according to the paradormancy processes described above. In male buds, the anthers remained in the microspore stem cell stage. On the same sampling date, ‘Franquette’ was at an earlier stage than ‘Chandler.’ In fact, ‘Franquette’ male buds did not reach the stage of development found in ‘Chandler’ on September 30, 2015, until the October 19, 2015 sampling date (Figure 6II). It should be noted that the reproductive structures had not undergone meiosis in any of the samples analyzed.

When comparing the evolutions described with those corresponding to September 23, 2016, in both cultivars, the development of female apical and lateral buds and male buds showed a greater advance than the buds analyzed on the same date of the previous year (Figure 8I). In the ‘Chandler’ male buds, the microsporangia of the anthers had immature pollen grains, which indicates that, unlike in September 2015, meiosis had already occurred. On October 5, 2016, only ‘Franquette’ was sampled (‘Chandler’ had already sprouted in the field), and it showed microsporangia in the anthers with microspore stem cells, although meiosis had not yet occurred.

At this point, it is interesting to contrast these results with observations in other fruit species. Julian et al. (2011, 2014), for instance, established that the development of stamens in apricot (*Prunus armeniaca*) continues during the autumn, and the flower buds enter dormancy with fully developed sporogenic tissue. Once the CRs are met and dormancy breaks, meiosis occurs. In walnut, Luza and Polito (1985, 1988) determined that the differentiation processes in staminate flowering must be completed after having met the winter CRs. Kvaliashvili et al. (2006) determined that meiosis occurs in walnut during the first half of April for different locations in the northern hemisphere (October in the South). In the present investigation, the atypical winter and spring thermal conditions of the 2016 season were responsible for the different meiosis dates in the cultivar ‘Chandler.’ In the winter of 2016, the chill accumulation reached 78.7 CP on July 31, while this number of CPs did not accumulate in the winter of 2015 until September 1. This explains our observations in the present investigation, in particular those of the cultivar ‘Chandler’ during the 2016 season. At the same time, the meiosis requirements of the cultivar ‘Franquette’ remain unclear, so it would be worth replicating the tests in successive years to validate the results obtained in this study. This would deepen our understanding of the interrelationships between the processes of accumulation of winter chill for dormancy release and spring heat for budburst and flowering in relation to the temporal occurrence of meiosis. These interrelationships are surely specific to the different cultivars studied.

Regarding the soluble sugar content, the dynamics of reserve sugars are linked to the dormancy-growth cycle and frost resistance in walnut and other temperate fruit species (Poirier et al., 2004; Charrier and Améglio, 2011; Guàrdia et al., 2016). Decourteix et al. (2006, 2008) and Alves et al. (2007) determined that there are proton-sucrose, proton glucose, and proton-hexose co-transporters in walnut xylem parenchyma cells and analyzed the potential role of these sugars in the events related to the start of spring growth.

The HPLC results for July 2015 in ‘Chandler’ showed similar concentrations of the three soluble sugars studied during endodormancy release in female buds and somewhat higher levels of sucrose in male buds. However, subsequent observations in the ecodormancy stage (08/19/2015; 09/02/2015) highlighted a sharp increase in sucrose, which reached maximum concentrations 15–30 days after dormancy breaking and then later fell abruptly as spring bud burst approached. Glucose and fructose, on the other hand, maintained stable concentrations or slightly lower amounts than those found in the endodormancy-release stage in both female and male buds. This partially disagrees with some previous results in the same species (Charrier et al., 2013, 2018) that describe uniform dynamics for the three sugars during this process. Similarly, Farokhzad et al. (2018), working with medium- and high-chill (i.e., late growth) walnut genotypes, showed that soluble sugars decreased during the dormancy release period. Nonetheless, the significantly greater increase in glucose in male than in female buds is in accordance with the lower chilling requirements of the male buds and with the characteristic protandry of ‘Chandler.’

At this point, it should be noted that in this experiment the CRs for bud dormancy release were fulfilled between July 12 and 31 in ‘Chandler’ and July 28 and August 12 in ‘Franquette,’ according to the observed year-by-year variability. After this period, substantial overchilling occurs during August in the region. Hence, the maintenance of high concentrations of sucrose may be associated with a cryoprotection function for the meristematic tissues of the buds. This would explain the differential regulation of sucrose with respect to the other hexoses and suggests that the role of this disaccharide cannot be extended to all the soluble sugars imported from the xylem sap, or, in the case of fructose and glucose, potentially formed by the dissociation of sucrose. In our analyses, the main role of sucrose during winter seems clear: it accumulates in buds for cold resistance functions, while both glucose and fructose contribute to a lesser extent to the carbon supply at this stage. Tixier et al. (2017) proposed a novel mechanism for the maintenance of the spring carbohydrate translocation in fruit trees from distal locations before the photosynthetic independence of the leaves and absence of transpiration. These researchers postulated that the flow of sugars through the xylem is maintained with the recirculation of water through the phloem, which acts as a hydraulic pump to generate a flow of water in the xylem, allowing for the transport and mobilization of sugars. In addition to this, glucose and fructose are more efficient osmotically than sucrose, so they may act as osmolytes to trigger water recirculation, increasing the vessel pressure needed for the growth of both female and male buds. The higher amounts of glucose and fructose observed in spring are likely involved in the carbon demand required for budding and flowering as determined by Maurel et al. (2004) in peach trees.

Yet, considering the high number of metabolites potentially involved in dormancy release, non-targeted metabolite studies could be more powerful than targeted ones to determine new metabolites associated with endodormancy release. Along these lines, Guillamón et al. (2020) have recently shown that endodormancy release is mainly driven by prunasin and ascorbic acid, which could be used as endodormancy release biomarkers. The application of non-targeted metabolite studies may open new avenues for the development of biomarkers for dormancy release and shed more light on the control of this complex process.

## Conclusion

We have presented a comprehensive dormancy progression study of two walnut cultivars, ‘Chandler’ and ‘Franquette,’ with markedly different CR. Our analyses of bud anatomical development and soluble sugar (sucrose, glucose, and fructose) dynamics in buds together with the artificial and field chill and heat trials provide an in-depth characterization of the dormancy release process in walnut in northern Patagonia.

The CR defined by artificial chill tests was higher than the field chill determinations. Moreover, the ‘Chandler’ cultivar required an average of 10.3 fields CPs (CP) and 2,163 Growing Degree Hours (GDH°C) less than ‘Franquette’ for dormancy release and bud burst, respectively. HR was the main driver in the phenology dynamics, as expected for a high-chill region like northern Patagonia.

These results were consistent with the anatomical and morphological changes observed in the shoot apex by light microscopy and the sugar profile determined by HPLC. The transition after dormancy release from microsporangia with sporogenous tissue to microspore mother cells supports the chilling requirement definition and the different CRs of the ‘Chandler’ and ‘Franquette’ cultivars. The decrease in sucrose 15–30 days after CR fulfillment could be a reliable biological marker for the transition from endodormancy to ecodormancy in walnut, while the increase in fructose and glucose is likely involved in the carbon demand required for budburst and flowering. In our experimental conditions, anther meiosis in ‘Chandler’ was observed in pre-sprouting. In addition, we have discussed the effects of paradormancy thanks to our non-decapitated/decapitated shoot experiment. Both cultivars showed signals of correlative inhibition processes among apical and lateral buds throughout dormancy progression.

Our results improve the current understanding of endo-ecodormancy progression in walnut and provide insightful results for walnut production and breeding. We provide a multi-year evaluation of the chilling requirements for the most representative cultivars used in northern Patagonia. Knowledge of a given cultivar’s chilling requirements and heat accumulation is key in selecting the most optimum cultivar for the most suitable areas. Regarding cultivation practices, late pruning is recommended to minimize the impact of spring frost. We have provided a complete data set for dormancy release for further application in dormancy modeling, which can be useful in inferring the ideotypes that should be bred for future climate conditions.

Finally, we have shown for the first time that anatomical and sugar dynamics support the definition of chilling and heat requirements in Persian walnut (*J. regia*) in northern Patagonia.

## Data Availability Statement

The raw data supporting the conclusions of this article will be made available by the authors, without undue reservation.

## Author Contributions

JC, GO, and RB conceived of the project. RB performed the experiments and measurements. AB collaborated in the analysis of results, and the creation of tables and figures. LL performed the microscope observations and analyzed the corresponding data. GO and CP analyzed the HPLC data. RB and JC wrote the manuscript with contributions from all the authors. All authors contributed to the article and approved the submitted version.

## Conflict of Interest

The authors declare that the research was conducted in the absence of any commercial or financial relationships that could be construed as a potential conflict of interest.

## Publisher’s Note

All claims expressed in this article are solely those of the authors and do not necessarily represent those of their affiliated organizations, or those of the publisher, the editors and the reviewers. Any product that may be evaluated in this article, or claim that may be made by its manufacturer, is not guaranteed or endorsed by the publisher.
